# Relational Quality of Registered Nurses and Nursing Assistants: Influence on Patient Safety Culture

**DOI:** 10.3390/healthcare9020189

**Published:** 2021-02-09

**Authors:** Amy Campbell, Diana Layne, Elaine Scott

**Affiliations:** 1 College of Nursing, East Carolina University, Greenville, NC 27858, USA; SCOTTEL@ecu.edu; 2 College of Nursing, Medical University of South Carolina, Charleston, SC 29425, USA; layne@musc.edu

**Keywords:** patient safety, relational quality, registered nurse, nursing assistants, teamwork, communication

## Abstract

Registered nurses (RNs) working within acute care hospitals have an incredible responsibility to provide safe care in a complex environment which requires trust, teamwork, and communication. Nursing assistants (NAs) play a critical role in working with RNs to meet these growing demands of inpatient care. Minimal evidence exists exploring the relational quality between RNs and NAs within hospitals. The aim of this study is to explore RN and NA behaviors and experiences that promote patient safety and teamwork and enhance communication between RNs and NAs within the hospital environment. Qualitative analysis was used, with two focus groups which included six participants within each group (three RNs and three NAs) from two separate inpatient units. Transcripts were reviewed and coded for themes. Collaborative teamwork and two-way communication were commonly reported as behaviors that promote patient safety. Trust between RNs and NAs was identified as a key component of positive relationships between RNs and NAs. Participants identified four common behaviors that build trust, which were accountability, effective conflict resolution, collaborative teamwork, and prioritizing patient needs. Finally, teamwork was identified as a common strategy to increase communication effectiveness between RNs and NAs. High relational quality (RQ) between the RN and NA is an important component of teamwork and patient safety culture.

## 1. Introduction

In today’s complex healthcare environment, the registered nurse has a tremendous responsibility to care for patients and manage the physical and emotional stress that accompanies their role. These stressors include increased acuity, short staffing, and working long hours away from their family [1,2]. To lessen the burden on the registered nurse (RN), the nursing assistant role was developed to serve as an extension of the RN. As an unlicensed professional, the nursing assistant (NA) works with the RN to assist patients with daily care, such as ambulating, bathing, and feeding. Other typical responsibilities of the NA include collecting vital signs as well as other tasks delegated by the RN. In some cases, this decreases the job satisfaction for the RN [3]. The RN is ultimately responsible for all the care provided by the NA as well as patient outcomes. Role confusion and differences in mental models and educational levels create barriers to teamwork and communication [4,5,6]. Ultimately, the inability to communicate, delegate, and work as a team can lead to poor outcomes for the patient [3].

The importance of relationship quality is discussed within the literature in the context of organizational effectiveness and teamwork [3,7,8]. The relational quality between RNs and NAs also has a correlation with patient safety [9]. The purpose of this qualitative research study is to explore RN and NA relational quality (RQ) and examine behaviors and experiences that promote patient safety and teamwork and enhance communication between RNs and NAs within acute care.

## 2. Materials and Methods

### 2.1. Recruitment

Following approval by the University and Medical Center IRB (19-000259), RNs and NAs from the highest and lowest relational quality units within an acute care system were approached for participation in this study. To determine these respective units, relational quality was measured for RNs and NAs across 53 inpatient units within the healthcare system. An initial study tested the adaptation of the Leader Member Exchange-7 instrument (LMX-7) [10] for use with RNs and NAs. Development of this instrument has been described elsewhere [11]. Data collected were used to identify the inpatient units with the highest and lowest relational quality. Based on these findings, face to face and email recruitment was conducted by a team member from the hospital’s Center for Research and Grants on selected units. A purposive sample of three RNs and three NAs was recruited from each unit for participation in a 60-min focus group. Participants received a 25-dollar gift card following participation in the focus groups. This study builds on the initial study to compare and contrast the relational quality characteristics of RNs and NAs within the high and low scoring units.

### 2.2. Data Collection

A qualitative exploratory-descriptive design was implemented with focus groups in September 2019. This was the most appropriate design to explore perceptions of registered nurses and nursing assistants related to experiences that promote patient safety and teamwork and enhance communication among this dyad. Qualitative research is most often used to examine beliefs, attitudes, behaviors, and experiences of participants [12]. Recruited participants attended one of two focus groups based on whether they worked on the unit with high relational quality or the unit with low relational quality. The principal investigator was blinded to the relational quality and unit of participants. Discussions were moderated by the first author (AC), a research mentor (ES), and the co-investigator (DL) and occurred in two separate conference rooms within the study site. Conference rooms were not located within patient care areas of participants to maximize confidentiality. A semi-structured interview guide was used which included the following questions: (Q1) “How do you work with team members on your unit to assure patient safety?”; (Q2) “How does the quality of the RN-NA relationship influence your everyday work in caring for a group of patients (you can share good and bad influences)?”; (Q3) “What behaviors (from an RN or NA depending on the participant) help you build trust in that relationship?”; (Q4) “What makes communication between the RN-NA more effective in assuring patient safety?”. Discussions were recorded with a digital audio recorder, professionally transcribed, and reviewed by the first author and co-investigator for accuracy. Researchers utilized field notes during each focus group.

### 2.3. Data Analysis

Inductive content analysis was utilized to analyze the focus group transcripts to transform the data from individual descriptions into a meaningful interpretation. The aim of inductive content analysis is to derive coded categories directly from the text, which is helpful when existing evidence studying the phenomenon is lacking [13]. There are limited previous studies examining the relationship between the registered nurse and nursing assistant dyad, which is what prompted the decision to utilize inductive content analysis [14]. Each transcript was independently coded by the first and second author in an effort to condense the data into analyzable units using Microsoft Word. Codes were assigned a priori (e.g., questions from the interview guide) or as emergent themes. The project team met and came to consensus on a final list of themes. Themes were then organized by relational quality group and categorized by strategies and behaviors to ensure patient safety, relational quality, trust, and effective communication.

## 3. Results

### 3.1. Characteristics of Both Relational Quality Groups

In both groups, the RNs and NAs reported collaborative teamwork and two-way communication as important strategies to assure patient safety. An RN participant from the high group shared, “To me, it [team turns-turning the patient with an RN and NA] just pretty much promotes team enhancement, and so to speak. It just kinda gives that nurse and care partner an opportunity to collaborate. But to me, it gives you some type of engagement.”. One NA participant from the low group shared “Because if I have a nurse that gives me attitude, I’m not going to be as quick to do for her and to communicate with her as I would somebody who respects me as much as I respect them and helps me as much as I’d help them.” An unintended consequence of poor relational quality between this dyad may be a lack of two-way communication.

In addition, positivity was stated as a strong influence to promote constructive relational quality. Trust was also reported as another essential component which influenced the quality of the RN-NA interaction. Both groups found effective communication, collaborative teamwork, and prioritizing patient needs as influences on trust. Teamwork was identified as a strategy that increases communication effectiveness between RNs and NAs in both RQ groups. Both high and low RQ groups displayed positive body language and discussed the importance of always putting the patient first.

### 3.2. Characteristics of the High Relational Quality Group

The high relational quality group displayed professional etiquette from the beginning of the interviews. They nodded while others were talking, encouraged each other to participate, and smiled a lot. Often the RN would stop and ask the NA their thoughts and perceptions. They encouraged each other to speak freely, and there was obvious psychological safety. To ensure patient safety, the high RQ group shared examples of team engagement “We’ve implemented team turns, and that’s every two hours you have a RN and care partner to do turns on a patient that requires two hour turning,” reported an RN participant. In building relational quality an RN stated, “I think for the most part, from what I’ve seen, on our unit, we tend to really be a team. We try to embrace any people that are new, or somebody may have been transferred from another unit to our floor, to try to provide them support.” An NA discussed the importance of respecting expertise to foster a strong relationship, “We were getting a lot of new nurses and they really don’t know, or they are afraid of what they might find, versus the older care partners that’s been there a long time, they can detect if something really going on with the patient.” To foster trust, the high RQ team focused on mindfulness and professionalism in building trust. Examples of trust from an RN participant: “I would say communication. Being open and [having an open] tone. How you approach someone, how do you talk with them? It has a professional level but also an assertive level.” In addition, participants reported the importance of recognizing various styles of communication and supportive listening as strategies to improve communication between the RN and NA. An example from the high RQ group:

So the communication again is like the main thing and then [you] know how to plan your day based on the reports you’re given. Cause some patients are heavier load [work intensity], some are a little bit lighter loads [work intensity]. You kind of can know when that care partner may need more help with that particular patient.

### 3.3. Characteristics of the Low Relational Quality Group

Participants within the low relational quality group reported other essential tasks for patient safety including bedside shift report, rounds, and visual cues. There was a sense of familiarity within the team. They spoke over each other, interrupted, used profanity, and would disagree while others were talking. One RN described the importance of visual cues as “also a cue, like if she goes into my room she knows, ‘Oh they’re NPO [nothing by mouth]. Oh, we need to record their output’.” The low RQ group discussed positive relationships, “But if you have good people and you have a good rapport with them and a good relationship with them and you can cut up and you can joke and you can find some laughter in the chaos of a 12 h shift, it makes that shift a little bit more bearable and it makes taking care of somebody an easier job.” Participants from this group reported additional obstacles including perceptions related to job demands and stress. The low RQ team found appreciation, prioritizing of patient needs, and fair distribution of work as positive influences on relational quality. Mental and physical exhaustion, burnout, and lack of accountability were negative influences on trust building. One participant stated,

I think people tend to get in this field [healthcare], they get burnt out. I think people get in this field [healthcare] and they tend to forget where they’ve come from because at the end of the day, you’re lettering degree, certifications] behind your name and the money that you make on your check is not going to mean anything when you’re six feet deep.

Meanwhile, participants from the low RQ group described trust as “Being honest with each other. Yeah, knowing when to ask for help and calling somebody on their bad behavior.” The low relational group found teamwork to be an influencer on communication. An example from an NA in the low RQ group:

I think it’s in your approach. Like I know I’m not going to go over to her and be like, ‘Hey, I need you to do this for me’ because she’s going to be like ‘Uh, what?’ You know, it’s like, ‘Hey, can you help me do this?’ and then if she’s going to go in and give a bath, I’m going to go in with her because it’s going to get done quicker and then we can assess the patient’s back side together and I can do a dressing change while we’re in there and stuff like that. So, it’s really in your approach throughout the whole day because it’s not our job as nurses to boss people around, it’s our job to work people as a team. You’re a team, do it together. That’s what I do at least.

Participants within this group also reported the importance of effective delegation: “Well a patient wanted to come out of his two points [restraints] and he said, ‘Can you take me out [of restraints]?’ and I said, ‘I can’t take you out’ and I said ‘That has to be delegated to me by my nurse’, I said ‘And it has to be delegated to her by the doctor’.”

## 4. Discussion

### 4.1. Mindful Interactions

In the high relational quality group, they focused on mindfulness to foster professional relationships. This was demonstrated by the body language, being in the moment (off their phones), and respect for one another while speaking. The participants showed up together, shook hands, and expressed gratitude for the opportunity to provide feedback. They would pause before speaking to best articulate their thoughts. They would repeat questions to validate and verify they understood the question in addition to the comments made by the other participants. In the low RQ group, they arrived one by one and were often reactionary in their comments. The term mindfulness has been defined as the ability to assign certain emotions and attention in the present moment [15]. Davies [16] discusses mindfulness as a strong weapon to regulate emotions and prevent burnout. Mindfulness has been found to benefit the individual’s wellbeing, professional relationships, and patient outcomes [17].

Leaders have a unique opportunity to foster and role model mindfulness within their teams. One strategy is to address issues in the moment, listening and seeking conflict resolution. There are many reasons conflict can arise between the RN and NA including educational differences, cultural differences, or generational differences. It is important for the leader to be mindful of biases that may exist related to educational, cultural, and generational differences between the RN and NA dyad. These biases may result in conflict within the RN-NA dyad. Unmanaged conflict can be a threat to patient safety [18]. In both RQ groups, participants described the importance of strong leadership, specifically to address unprofessional behaviors and actively work to mitigate safety issues related to patient care.

### 4.2. Self-Awareness

In the low relational quality group, participants seemed to display a lack of professionalism and a familial relationship instead of a professional relationship between the RN and NA. There is a danger with familiarity. Familiarity can breed contentment and can lead to unhappiness and unrealistic expectations [19]. Familiarity can also be a contributor to burnout, exhaustion, and siloed work [20]. Working in silos jeopardizes safety, decreases efficiency in care, and decreases patient satisfaction [21]. During the focus group, the participants in the low RQ group stated burn out was a barrier to relational quality. The NAs often felt they were working in silos. This broken system decreases the resilience of the nurses [22] (contributing to the mental and physical exhaustion of staff. As leaders, we have to recognize the potential for these silos between the RN and NA and encourage teamwork to increase patient safety and ensure relational quality.

### 4.3. Self-Reflection

In both groups, there was concern about the manager’s role in addressing behaviors and resolving conflict. Calling behaviors early can set the team members up for success. Unresolved conflict can lead to turn over, dissatisfaction, and possible litigation. It is important to deal with conflict early and to seek solutions to the problem. As managers, one should consider how to be purposeful in rounding to help build trust and establish rapport and accountability in a stressful environment. The manager should consider cultural, generational, and educational barriers. These barriers can be addressed in professional development. Simulation and role play are strategies managers may find helpful to assist the RN-NA dyad in building and maintaining relational quality [23].

## 5. Conclusions

Relational quality is critical to patient safety [3]. The RN and NA spend the most time providing direct patient care and need to be able to work together as a team to achieve the best outcome for the patients [24]. The manager has a role in the development of this team, ensuring this dyad can work together, communicate effectively, and prioritize patient needs. Mindfulness, self-awareness, and self-reflection are important in any relationship, especially in healthcare. Consideration for incorporating specific training, such as role play, and simulation into educational preparation for both RNs and NAs may also improve relational quality among this dyad. With rapidly changing healthcare environments, nurse leaders need to focus on fostering strong relational quality between the RN and NA to maximize patient safety.

## Data Availability

Data available on request due to privacy restrictions.
